# ﻿New data on bioacoustics and courtship behaviour in grasshoppers (Orthoptera, Acrididae, Gomphocerinae) from Russia and adjacent countries

**DOI:** 10.3897/zookeys.1200.118422

**Published:** 2024-05-02

**Authors:** Varvara Vedenina, Nikita Sevastianov, Evgenia Kovalyova

**Affiliations:** 1 Institute for Information Transmission Problems, Russian Academy of Sciences, Bolshoy Karetny per. 19, Moscow 127051, Russia Institute for Information Transmission Problems, Russian Academy of Sciences Moscow Russia

**Keywords:** Calling song, courtship song, frequency spectrum, stridulatory leg movements, visual display

## Abstract

The songs of seven grasshopper species of subfamily Gomphocerinae from Russia, Ukraine, Georgia, and Kazakhstan were studied. We analysed not only the sound, but also the stridulatory movements of the hind legs to more entirely describe the songs. In *Mesasippuskozhevnikovi*, *Chorthippusmacrocerus*, and *C.hammarstroemi*, the legs are moved in a relatively simple pattern; four other species, *Myrmeleotettixpalpalis*, *Stenobothrusnewskii*, *C.pullus*, and *Megaulacobothrusaethalinus* demonstrate more complex leg movements. In six of the seven species studied, the courtship songs contain more sound elements than the calling songs. The highest number of courtship sound elements was found in *M.palpalis* and *M.aethalinus.* The different parts of a remarkably long stridulatory file in *M.aethalinus* are thought to participate in the production of different sound elements. The songs in *S.newskii* are shown for the first time. This species produces sound not only by common stridulation but also by wing beats. A relationship of *S.newskii* to some other species of the genus *Stenobothrus*, which are able to crepitate, is discussed. We also analyse the frequency spectra of the songs. A maximum energy of the song power spectra in six species studied lies in ultrasound range (higher than 20 kHz). In only *M.aethalinus*, the main peaks in the song power spectra lie lower than 20 kHz. The courtship behaviour in *M.palpalis*, *C.macrocerus*, and *C.hammarstroemi* was shown to include conspicuous visual display (movements of antennae, palps and the whole body).

## ﻿Introduction

In many species of Orthoptera, the song is an important component of reproductive isolation. This is the reason why acoustic signals are often used in taxonomy, when sibling species are similar in morphology, but have quite different songs. Among the Acrididae subfamilies, acoustic communication in Gomphocerinae is most developed in terms of structure of acoustic apparatus, temporal pattern of the song, and mating strategies (e.g., Otte 1970; von Helversen and von Helversen 1994; Ragge and Reynolds 1998). The song is produced by stroking a stridulatory file on each hind femur across a raised vein on the fore wing. Using both hind legs, the grasshoppers have two separate sound-producing devices that must be coordinated with one another. The stridulatory movements of the two legs often differ in amplitude and form, and the legs can exchange roles from time to time, leading to increased song complexity (Elsner 1974; von Helversen and Elsner 1977; Elsner 1994). Various species demonstrate different degrees of song complexity. The song in Gomphocerinae also varies according to the behavioural situation. A solitary male produces a calling song, listening for the response song of a female that is ready to mate. Several males sitting in a close vicinity can produce rival songs. When a male finds a female, in many species the male begins a special courtship song, which may reach a high complexity and may be accompanied by conspicuous movements of different parts of the body such as the abdomen, head, antennae, or palps (Faber 1953; Otte 1970; von Helversen and von Helversen 1994).

To make a comprehensive analysis of songs between the species, it is necessary to compare not only the sound but also the stridulatory leg movements. Sometimes, a similar sound pattern can be produced by completely different leg movements (Vedenina and Mugue 2011). The leg movement analysis may help in the sound analysis when the gaps between sound elements are not distinct because of the phase shift between the two legs. A comparison of the leg movements in different species rather than the sound analysis may indicate a relationship between the species (Sevastianov et al. 2023). During the courtship behaviour, a male may also demonstrate species-specific leg movements without producing the sound.

It was previously argued that the specificity of the Gomphocerinae songs lies not in their frequency band but almost without exception in the pattern of amplitude over time. However, several studies showed that despite a relatively broad spectra of the grasshopper songs, there are pronounced interspecific differences in maxima or peaks (Meyer and Elsner 1996, 1997). It was also shown that male calling and female response songs may differ in the frequency spectra, and these differences can be used during species recognition (von Helversen and von Helversen 1997). It was also shown that various parts of elaborate courtship songs may significantly differ in the carrier frequency (Vedenina et al. 2007; Ostrowski et al. 2009; Vedenina et al. 2020). The differences in the frequency spectra between the various song elements may influence the amplitude ratio on the oscillogram. If the song is recorded by portable recorders with a frequency range not exceeding 12.5–15 kHz, the amplitude ratio of different elements may be distorted (Vedenina and Shestakov 2014).

In the current paper, we describe the calling and courtship songs in seven species of Gomphocerinae from Russia, Ukraine, Kazakhstan, and Georgia. To gain a better description of the songs, we analyse not only the sound, but also the underlying stridulatory movements of the hind legs. We also consider the whole visual display accompanying the courtship song in some species. And finally, we analyse the frequency spectra of the songs and different song elements.

## ﻿Materials and methods

The calling song was recorded from a solitary male; the courtship song was recorded when a male was sitting near a female. All song recordings were made in the laboratory. Both the sound and the movements of the hind legs were recorded with a custom-built opto-electronic device (von Helversen and Elsner 1977; Hedwig 2000). A piece of reflecting foil was glued to the distal part of each hind leg femur of the male and two opto-electronic cameras were focused on the illuminated reflecting dots. Each camera was equipped with a position-sensitive photodiode that converted the upward and downward movements of the hind legs into voltage signals. These signals, together with the recordings of the sounds (a microphone type 4191, ½ inch; a conditioning amplifier type 2690; Brüel & Kjaer, Nærum, Denmark), were A/D-converted with a custom-built PC card. The sampling rate was 1325 Hz for recording the stridulatory movements and 100 kHz for sound recordings. The ambient temperature near the singing male was 30–32 °C. The temporal parameters and power spectra of the songs were analysed with COOLEDIT (Syntrillium, Seattle, WA) and TURBOLAB 4.0 (Bressner Technology, Gröbenzell, Germany). Courtship behaviour was also recorded with a Sony HDR-CX 260E digital video camera; the video signals were analysed with the VIRTUAL DUB program.

**Figure 1.**
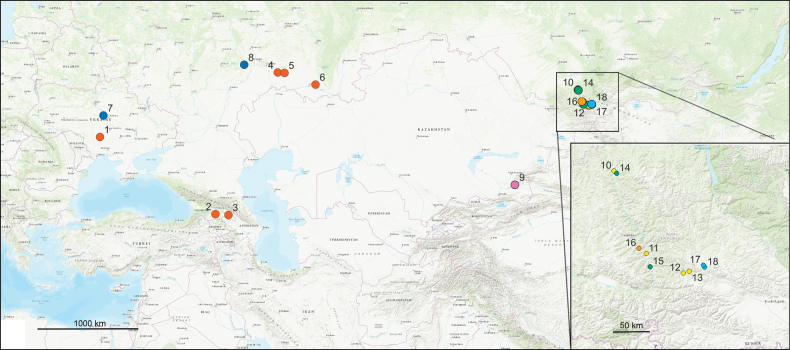
Map of localities where the specimens were collected for the song recordings. 1–6: *Chorthippusmacrocerus*; 7, 8: *Chorthippuspullus*; 9: *Mesasippuskozhevnikovi*; 10–13: *Megaulacobothrusaethalinus*; 14, 15 *C.hammarstroemi*; 16: *Myrmeleotettixpalpalis*; 17, 18: *Stenobothrusnewskii.* The localities of the same species are indicated by the same colour.

Localities where the song recordings were made are shown in Fig. 1. The numbers of localities in the text (paragraphs Material in description of each species) correspond to the numbers on the map. Data on species distribution were obtained from Bey-Bienko and Mistshenko (1951) and Ragge and Reynolds (1998).

**Figure 2.**
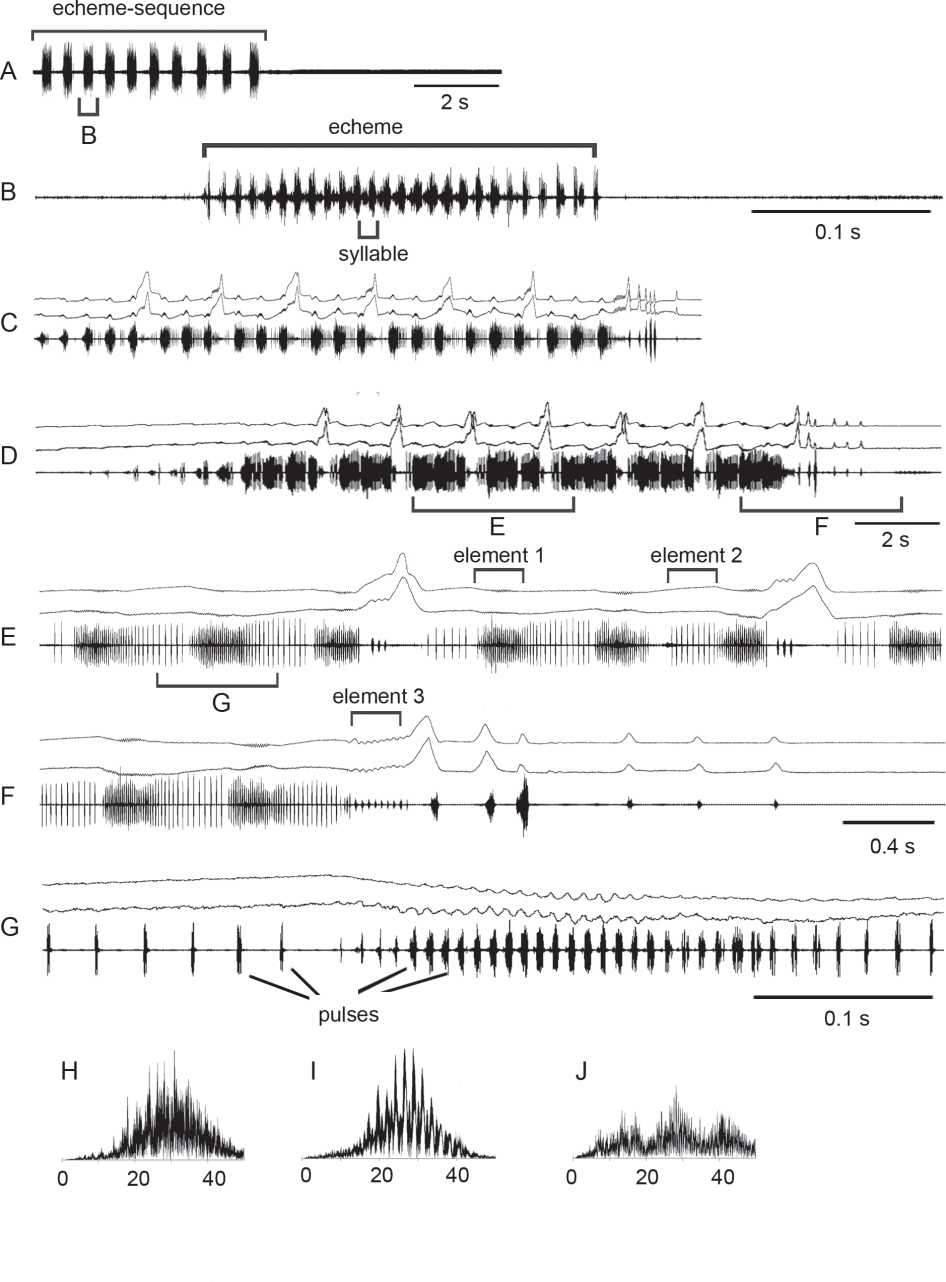
Oscillograms of the calling song **A, B**, courtship songs **C–G** and frequency spectra **H–J** in *Myrmeleotettixpalpalis*. Courtship songs of two males are shown in **C** and **D**. Song recordings are presented at three different speeds. In oscillograms **C–G** the two upper lines are recordings of hind leg movements and the lower line is the sound recording. Different elements of the courtship song are shown in **E, F**. Frequency spectra are shown in kHz for the courtship elements 1 **H**, 2 **I** and 3 **J**.

For the song description we used the following terms: **pulse** – the sound produced by one stroke of a hind leg and representing the shortest measurable unit; **syllable** – the sound produced by one complete up and down movement of the hind legs, starting when the legs leave their initial position and ending when the legs return to their original position; **element** – the sound produced by the same leg movements and usually including a series of equal syllables; **echeme** – series of consistent syllables separated by pauses (Fig. 2).

## ﻿Results and discussion

### ﻿ Myrmeleotettix palpalis

Taxon classificationAnimaliaOrthopteraAcrididae

(Zubowsky, 1900)

FE0D4EB4-6C99-5797-B76C-F3C391F3ED21

#### Distribution.

Southern Siberia from Altai to Transbaikalia, south-west of Amur region to Mongolia. Abundant in dry steppes and semi-deserts.

#### Material

**(Fig. 1).** 16. Russia: Altai Republic, ~ 26 km SE of Ongudai, environs of Kupchegen’, 50°37.3'N, 86°26.2'E, 922 m a.s.l., 05.08.2023, song recordings in 3 ♂.

#### References to song.

Tishechkin and Bukhvalova 2009: recordings of calling song from Buryatia and Chita region.

#### Song.

The calling song is an echeme-sequence lasting for ~ 7 s and consisting of ~ 14 echemes (Fig. 2A–B). The echemes usually lasts ~ 0.2–0.25 s and the intervals between them are ~ 0.3–0.4 s. Each echeme consists of ~ 25 pulses repeated at the rate of 120–125 /s. Oscillograph analysis shows that the low-amplitude pulses are sometimes produced in gaps between the main pulses.

In the courtship song, one can distinguish three sound elements (Fig. 2C–J; see Suppl. material 1). The element 1 is similar to the calling echeme, although with clear gaps between pulses. The pulses are produced by synchronous leg movements repeated at the rate of ~ 115–120 /s. Each pulse is generated by only downstroke. The element 1 gradually transforms to element 2: the rate of pulses decreases to ~ 40–50 /s and they become ~ 2 × as long. It is remarkable that they are produced by very weak up-movement of one leg. After three alternations of elements 1 and 2, a male produces a high-amplitude stroke with both legs, despite two legs produce different patterns. One leg is moved up in a stepwise manner, which results to generation of 3–7 low-amplitude pulses (element 3), whereas another leg is moved up straighter without low-amplitude vibrations. Then elements 1 and 2 alternate again 3×, followed by the high-amplitude stroke of the legs, which change the roles. After repeating ~ 6–7 cycles with high-amplitude strokes, the two legs are moved synchronously producing element 3, followed by precopulatory leg movements. The frequency spectra of elements 1 and 2 are similar, occupying a broad range from 15 to 40 kHz with maximum energy between 25 and 35 kHz (Fig. 2H, I). The spectrum of element 3 has three maxima at ~ 15, 30, and 40 kHz (Fig. 2J).

**Figure 3.**
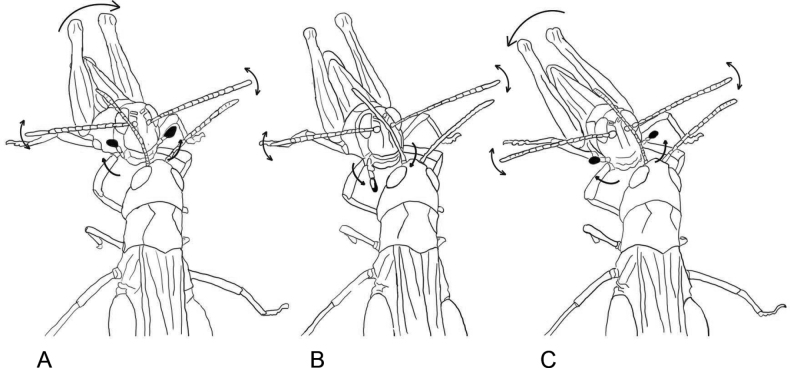
Movements of antennae, black and white palps and the whole body in *Myrmeleotettixpalpalis* during courtship. The palps are raised up **A** lowered down **B** and raised up again **C**.

When producing alternation of elements 1 and 2, a male slightly moves his body from side to side, generates low-amplitude movements with antennae keeping them turned to the sides, and conspicuously moves with black and white palps up and down (Fig. 3).

#### Comparative remarks.

The recordings of calling song in *M.palpalis* from Altai are similar to the recordings from Buryatia and Chita region (Tishechkin and Bukhvalova 2009). The courtship song of *M.palpalis* is shown for the first time. The calling song and the courtship element 1 are produced during the leg movements repeated at the rate of ~ 115–120 /s. According to Vedenina and Mugue (2011) and to Sevastianov et al. (2023), this pattern can be considered as relatively complex and advanced pattern: the leg-movement rate could originate from the double rates of the wing beat. The complex structure of the courtship song with several sound elements found in *M.palpalis* are concordant with the overall complexity of the courtship songs in the genus *Myrmeleotettix*. (Ragge and Reynolds 1998; Berger and Gottsberger 2010; Vedenina and Mugue 2011; Vedenina et al. 2020).

We suggest the movements with palps to be a remarkable visual display that distinguishes *M.palpalis* from most gomphocerine species. In one more species of this genus, *M.antennatus*, the palp movements were also described during courtship (Berger and Gottsberger 2010). *M.antennatus*, however, moves palps much more rapidly than *M.palpalis*, and the most conspicuous visual display in *M.antennatus* comprises a large swing of antennae with well-developed clubs. In contrast to *M.palpalis*, pulps in *M.antennatus* are not coloured in black and white. Within the genus *Myrmeleotettix*, antennae in *M.palpalis* are least thickened at the ends, which is probably correlated with very weak antennal movements during courtship. Other two species of this genus, *M.maculatus* (Ragge and Reynolds 1998; Vedenina and Shestakov 2014) and *M.pallidus* (Vedenina et al. 2020) move conspicuously with antennae but not with palps. Thus, the visual display in various *Myrmeleotettix* species seems to evolve independently. The palp movements are also known in *Aeropussibiricus* and *Gomphocerippusrufus* (e.g., Elsner 1974; Ragge and Reynolds 1998). However, these species that are distantly related to the species of *Myrmeleotettix*, demonstrate very different patterns of the palp movements. It is evident that the pulp movements in *Myrmeleotettix*, *A.sibiricus*, and *G.rufus* evolved convergently.

### ﻿ Stenobothrus newskii

Taxon classificationAnimaliaOrthopteraAcrididae

Zubowsky, 1900

3FFF7DCA-F3EF-58E3-AE01-1222D92A2B0D

#### Distribution.

Altai Mountains, Tuva, NW Mongolia. Usually associated with alpine meadows.

#### Material

**(Fig. 1).** Russia: 17. Altai Republic, Ulagan district, 3.5 km N of Lake Cheybek-Kohl, 50°25.854'N, 87°34.561'E, 1907 m a.s.l., 06.08.2023, song recordings in 3 ♂; 18. Altai Republic, Ulagan district, ab. 10 km N of Aktash, near Lake Cheybek-Kohl, 50°24.5'N, 87°35.8'E, 1821 m a.s.l., 14.08.2021, song recordings in 5 ♂, 06.08.2023, song recordings in 3 ♂.

#### References to song.

Unknown.

#### Song.

**Figure 4.**
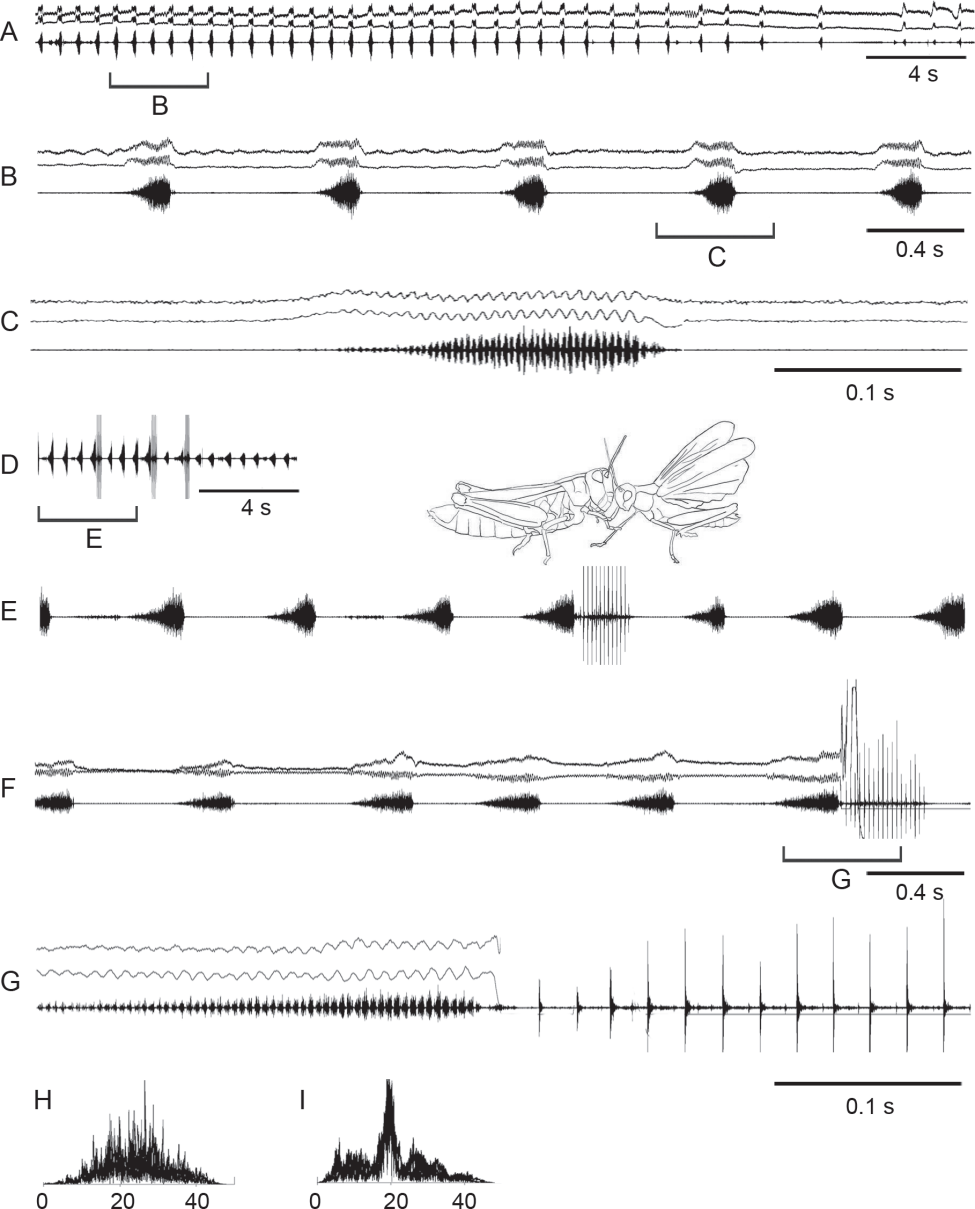
Oscillograms of the calling song **A–C** courtship song **D–G** and frequency spectra **H, I** in *Stenobothrusnewskii*. Courtship songs of two males are shown in **E** and **F**. Song recordings are presented at three different speeds. In oscillograms **A–C, F–G** the two upper lines are recordings of hind leg movements and the lower line is the sound recording. The drawing shows the wing clapping, which generates the high-amplitude pulses shown in **D–G**. Frequency spectra are shown in kHz for the main echeme **H** and wing beats **I**.

The calling song is an echeme-sequence that may last for tens of seconds, up to a minute (Fig. 4A–C). The echemes usually lasts ~ 0.25 s and the intervals between them are ~ 0.6 s. Each echeme begins quietly, reaching maximum intensity at the second half of its duration. Each echeme is generated by the low-amplitude, antidromic up and down leg movements at the rate of ~ 120 /s. During both up and down movements, the legs produce distinct pulses so, that the pulse rate is 2× as high as the leg-movement rate.

During courtship, the males generate a sequence of echemes almost identical to the calling echeme-sequence. However, sometimes a courting male shortly crepitates by wings (Fig. 4D–G; see Suppl. material 2) or / and starts a noisy flight. After such a flight, the male is trying to copulate. The frequency spectrum of crepitation has a rather narrow maximum around 20 kHz; by contrast, the spectrum of echeme is more usual for Gomphocerinae, ranging from 10 to 40 kHz with numerous maxima between 18 and 30 kHz.

#### Comparative remarks.

The acoustic behaviour in *S.newskii* is described for the first time. It is remarkable that this species crepitates in flight and is also able to generate short sequences of wing beats sitting on the ground. Such crepitation on the ground is also known in some other species of the genus *Stenobothrus*, namely, *S.rubicundulus* (Elsner and Wasser 1995), *S.cotticus* (Berger et al. 2010), and *S.hyalosuperficies* (Tarasova et al. 2021).

The song and mating behaviour of *S.newskii* is almost identical to those in *S.cotticus* (Ragge and Reynolds 1998; Berger 2008; Berger et al. 2010). It is surprising considering the large distance between the localities of the two species. *Stenobothruscotticus* was originally assumed to be endemic to the southwestern Alps, and it was later found in the Rila mountains in Bulgaria (Berger et al. 2010). The authors suggested that *S.cotticus* had a wider distribution during colder periods, when its range was expanded to lower altitudes. It is remarkable that *S.newskii* is similar to *S.cotticus* not only in song but also in morphology (Zubowsky 1900; Kruseman and Jeekel 1967) and ecological preferences: both species occur in alpine meadows at altitudes higher than 1700 m a.s.l. Taking into account that the leg-movement patterns of both species are relatively complex and may be considered as evolutionary advanced (Vedenina and Mugue 2011; Sevastianov et al. 2023), we suppose that the two species may represent one taxon. However, this conclusion requires more confirmation.

### ﻿ Mesasippus kozhevnikovi

Taxon classificationAnimaliaOrthopteraAcrididae

Tarbinski, 1925

665CEF67-C888-5577-BAD7-AB9A962A66B3

#### Distribution.

Eastern and southern Kazakhstan, Uzbekistan.

#### Material

**(Fig. 1).** 9. Kazakhstan: Almaty region, national park Altyn-Emel’, environs of Basshi, along stream, 44°10.1'N, 78°45.1'E, 05.07.2016, song recordings in 2 ♂, 22.06.2023, song recordings in 3 ♂.

#### References to song.

Bukhvalova and Vedenina 1998: recordings of calling song from Kazakhstan.

#### Song.

**Figure 5.**
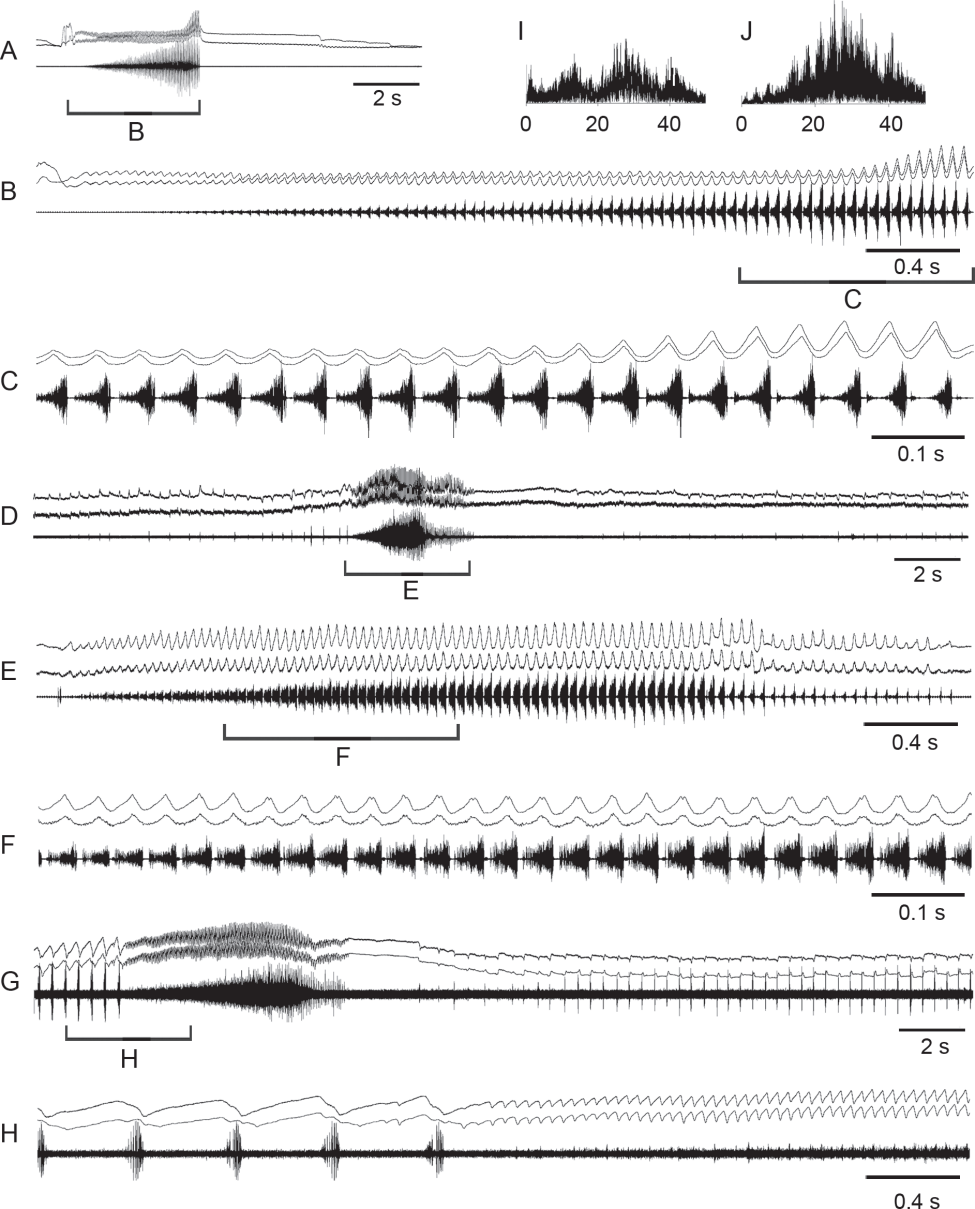
Oscillograms of the calling song **A, B** courtship song **C–H** and frequency spectra **I, J** in *Mesasippuskozhevnikovi*. Courtship songs of two males are shown in **D** and **G**. Song recordings are presented at three different speeds. In all oscillograms the two upper lines are recordings of hind leg movements and the lower line is the sound recording. Frequency spectra are shown in kHz for the short syllables **I** and the main echeme **J** of courtship.

The calling song is an echeme lasting for 3–4 s (Fig. 5). It begins quietly and reaches maximum intensity ~ 2/3 of its duration (Fig. 5A–B). The two legs are moved synchronously at the rate of 21–25 /s, generating homogenic syllables. During the upstroke, the legs generate a soft sound, whereas during the downstroke, the sound intensity gradually increases (Fig. 5C).

The courtship song starts with producing soft and short syllables, repeated at the rate of ~ 2–3 /s (Fig. 5G); however, sometimes these syllables are repeated very irregularly (Fig. 5D). When producing element 1, the legs are moved with a very small amplitude. In ~ 30 s–1 min a male starts an element 2 that is similar to the calling song. However, the echeme duration is longer, up to ~ 7–8 s. The echeme also begins quietly, reaching maximum intensity in ~ 2–4 s. The amplitude of the leg movements gradually increases but abruptly decreases close to the end; as a result, the syllables of smaller amplitude are generated at the end of each echeme. Then, the long element 1 is again produced, followed by element 2. The frequency spectrum of element 1 shows three maxima in a wide range (Fig. 5I), whereas the spectrum of element 2 is characterised by many peaks between 18 and 35 kHz (Fig. 5J).

#### Comparative remarks.

The current recordings of calling song are similar to the recordings published by Bukhvalova and Vedenina (1998), despite usage of the different recording equipment. The courtship song of *M.kozhevnikovi* is shown for the first time. According to Bey-Bienko and Mistshenko (1951), the localities where our material and that collected by Bukhvalova and Vedenina (1998) are situated within the range of subspecies *M.kozhevnikoviiliensis* Mistsh. In the genus *Mesasippus*, there are nine species occurring in Kazakhstan, Uzbekistan, western China, and north-western Mongolia. Currently, we have information on bioacoustics in only one species of the genus.

### ﻿ Chorthippus pullus

Taxon classificationAnimaliaOrthopteraAcrididae

(Philippi, 1830)

D2453D6C-0820-561B-B458-9739FA6B0DAD

#### Distribution.

Europe from France to the east of European Russia, reaching as far north as Leningrad region and as far south as the northern Caucasus. This species occurs very locally, either in mountains or in dry pine forests (sandy heathlands and forest clearings).

#### Material

**(Fig. 1).** 7. Ukraine: Cherkassy region, ~ 17 km S of Kanev, glades in pine forest, 49°35.58'N, 31°29.51'E, 22.06.2010, song recordings in 3 ♂; 8. Russia: Ul’yanovsk region, Novospassky district, Monastyrsky Sungur, 53°14.483'N, 47°39.839'E, 05.07.2022, song recordings in 2 ♂.

#### References to song.

Ragge and Reynolds 1998: recordings of calling song from Germany. Bukhvalova and Vedenina 1998: recordings of calling song from Ukraine, Zakarpat’je.

#### Song.

**Figure 6.**
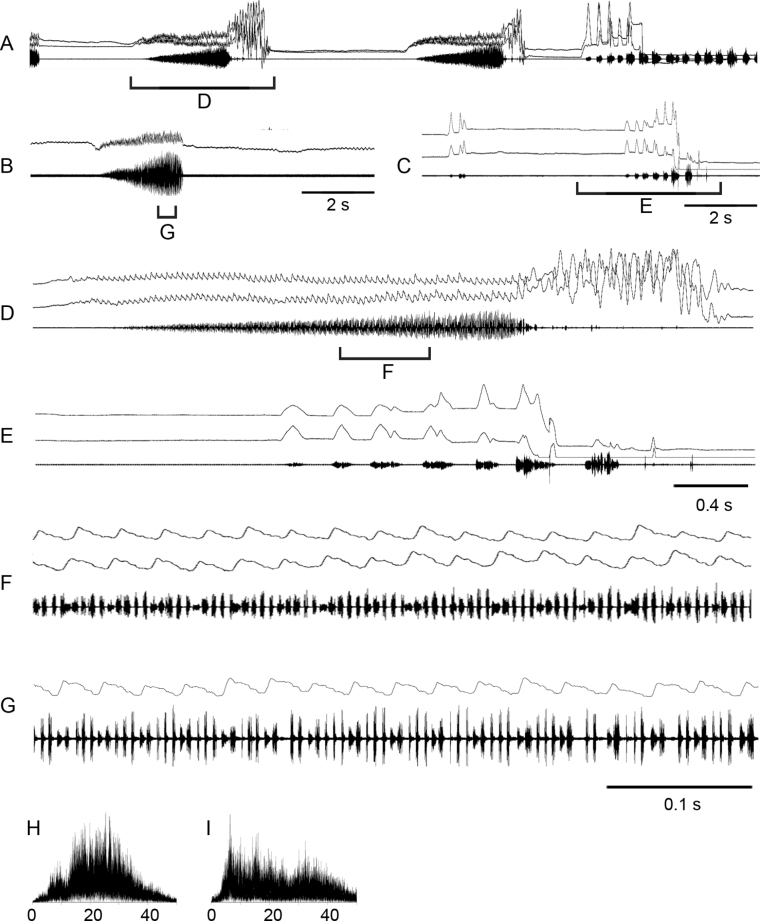
Oscillograms of the courtship song **A–G** and frequency spectra **H, I** in *Chorthippuspullus*. Courtship songs are shown in males from Ukraine **A, D, F** and Russia **B, C, E, G**. Song recordings are presented at three different speeds. In all oscillograms except **B, G** the two upper lines are recordings of hind leg movements and the lower line is the sound recording. Courtship song of one-legged male is shown in **B, G**. Frequency spectra are shown in kHz for the main echeme **H** and precopulatory sound **I**.

During courtship, a male generates several echemes each lasting ~ 2–4 s and repeated at ~ 3–4 s intervals (Fig. 6A). Each echeme has either one or two elements. The first element is a whizzing sound produced by the low-amplitude leg movements at the rate of ~ 34 /s. It begins quietly showing a gradual crescendo for approximately the first half of its duration. The two legs are moved with the notable phase shift (Fig. 6F). During the short up movement, each leg generates one soft pulse, whereas during stepwise down movement, each leg produces four pulses of increasing sound intensity. Thus, each syllable contains five pulses repeated at the rate of ~ 170 /s. Sometimes, immediately after the first echeme element, the legs are moved asynchronously with the high amplitude, which produces almost no sound (Fig. 6A, D; see Suppl. material 3). Before the copulation attempts, a male moves the legs synchronously with high amplitude generating several noisy syllables repeated at the rate of ~ 3.5 /s (Fig. 6A, C, E). The frequency spectrum of the main echeme is broad with maximum energy between 13 and 30 kHz (Fig. 6H); the spectrum of precopulatory sound has several maxima between 5 and 20 kHz (Fig. 6I).

#### Comparative remarks.

Ragge and Reynolds (1998) suggested sometimes two elements in the calling song of *C.pullus*, the first one being produced by the fast, low-amplitude leg vibrations and the second one by the slower, high-amplitude leg movements. We suggest that the second element mainly serves as a visual display during courtship, especially given the absence of the sound during the high-amplitude leg movements. Ragge and Reynolds (1998) also supposed that the leg movements producing the whizzing noise could be complex and asynchronous. We support their assumption by analysing the leg-movement pattern. The main rhythm of the leg movements (34 /s) could originate from the half rates of the wing beat (Vedenina and Mugue 2011). However, rapid vibratory movements during each downstroke (170 /s) are higher than the double rates of the wing beat. We consider the leg-movement pattern of *C.pullus* to be rare, if not unique, within Gomphocerinae.

The uniqueness of the leg-movement pattern of *C.pullus* within Gomphocerinae is in a concordance with its controversial taxonomic status. Despite this species is attributed to the genus *Chorthippus*, different phylogenetic reconstructions based on various molecular markers (Sevastianov et al. 2023; Schmidt et al. 2024) indicate that *C.pullus* forms an outgroup not only to the genus *Chorthippus*, but even to the tribes Stenobothrini and Gomphocerini. At the same time, morphologically this species could be easily attributed to *Chorthippus*, although being a rather brachypterous species.

### ﻿ Chorthippus macrocerus

Taxon classificationAnimaliaOrthopteraAcrididae

(Fischer-Waldheim, 1846)

B5A1FBD7-2EB9-563D-8778-D5B6ED368637

#### Distribution.

*Chorthippusmacrocerusmacrocerus*: Transcaucasia, Asia Minor, Iraq, northern Iran. *Chorthippusmacroceruspurpuratus* (Vorontsovski, 1928): from Ukraine to northern and western Kazakhstan, reaching as far south the northern Caucasus.

#### Material

**(Fig. 1).** 1. Ukraine: Nikolaev region, Pervomaisk district, Ostapovka, 47°58.2'N, 31°05.8'E, 05.07.2005, song recordings in 1 ♂; Georgia: 2. Algeti national park, 41°40.55'N, 44°21.55'E, 1252 m a.s.l., 27.08.2023, song recordings in 5 ♂; 3. environs of Sighnaghi, 41°35.91'N, 45°51.18'E, 770 m a.s.l., song recordings in 1 ♂; Russia: 4. Samara region, Alekseevka district, Gerasimovka, 52°42.636'N, 51°30.584'E, 12.07.2012, song recordings in 2 ♂; 5. Orenburg region, environs of Buzuluk, 52°40.7'N, 52°17.8'E, 30.06.2020, song recording in 1 ♂; 6. Orenburg region, Saraktash district, Studentzy, 51°51.639'N, 55°51.312'E, 14.07.2012, song recordings in 1 ♂.

#### References to song.

Vedenina and Zhantiev 1990; Bukhvalova and Zhantiev 1994; Vedenina and Bukhvalova 2001: recordings of calling song from Moldova, Ukraine, south-eastern part of European Russia and northern Caucasus.

#### Song.

**Figure 7.**
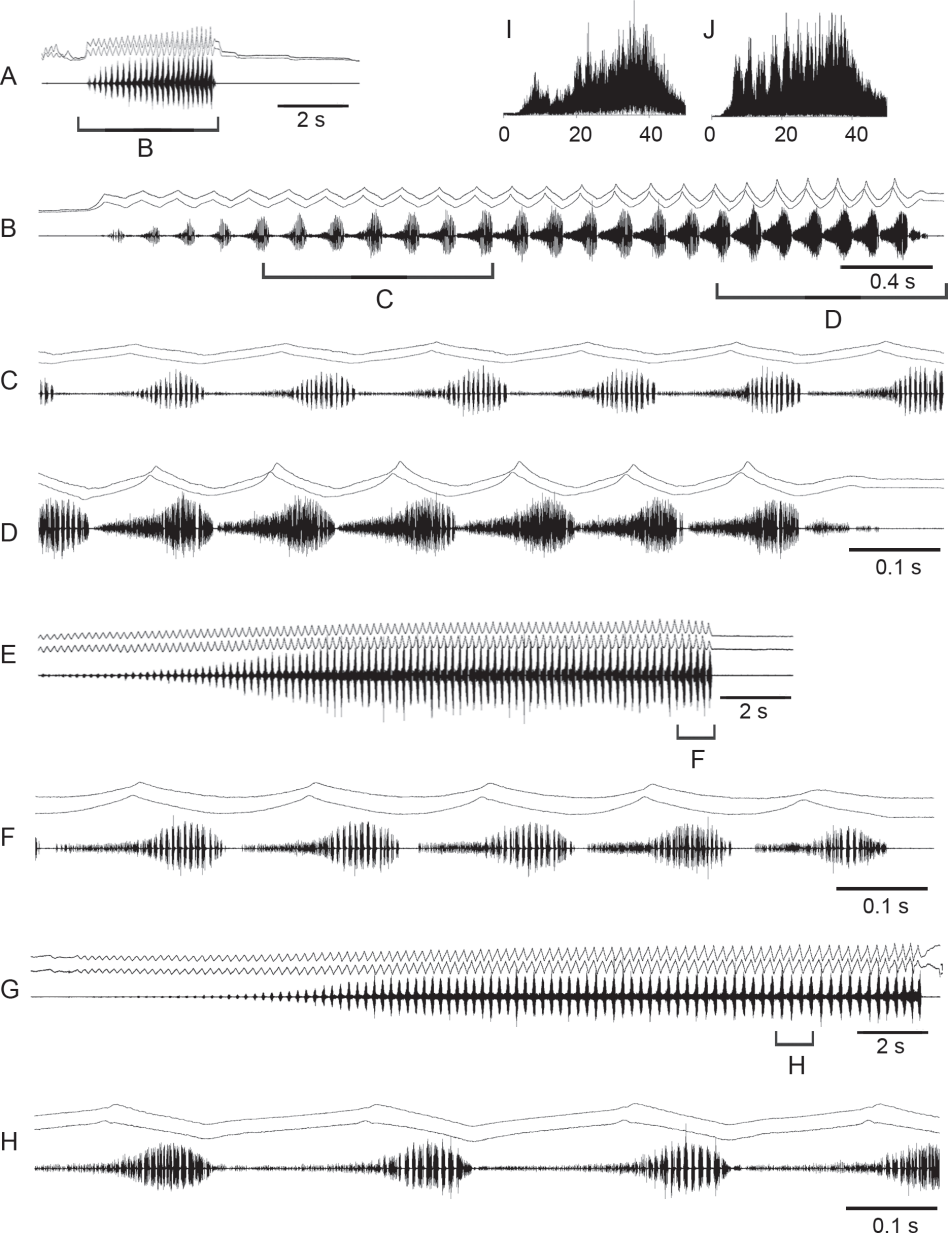
Oscillograms of the calling song **A–D**, courtship songs **E–H** and frequency spectra **I, J** in *Chorthippusmacrocerus*. Courtship songs are shown in males from Orenburg region **E–F** and from Georgia **G–H**. Song recordings are presented at three different speeds. In all oscillograms the two upper lines are recordings of hind leg movements and the lower line is the sound recording. Frequency spectra are shown in kHz for the courtship songs from Orenburg region **I** and from Georgia **J**.

The calling song is an echeme lasting 14–18 s in nominative subspecies, and shorter, ~ 4–10 s, in *C.macroceruspurpuratus* (Fig. 7). The legs are moved with a small phase shift at the rate of ~ 3.5–4.5 /s in nominative subspecies, and faster, at the rate of ~ 5–7 /s, in another subspecies. At the beginning of the echeme, each upstroke of the legs produces almost no sound, whereas each downstroke generates syllables of distinct pulses (Fig. 7C). Close to the echeme end, the legs produce a louder, but still relatively soft sound during each upstroke. Very often, the legs are moved faster close to the echeme end than at the beginning; as a result, distinct pulses disappear in the last syllables (Fig. 7D). This, however, occur only in *C.macroceruspurpuratus*. The nominative subspecies usually does not increase the rate of the leg movements close to the echeme end.

The courtship song is similar to the calling song but lasts longer. For example, in nominative subspecies the echeme duration varies in the range of 18–30 s (Fig. 7G). The frequency spectra are also similar in the calling and courtship songs. We, however, found some differences between the spectra of two subspecies. The spectrum of the *C.macroceruspurpuratus* song has the main maximum around 35 kHz but also a small peak around 10 kHz (Fig. 7I). The spectrum of the song in nominative subspecies has many periodical peaks in the broad range of 5–40 kHz (Fig. 7J).

**Figure 8.**
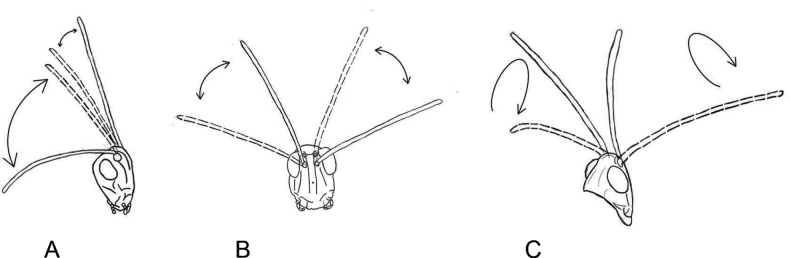
Movements of antennae during courtship in *Chorthippusmacrocerus*. Successive stages of the antennae position are shown in **A–C**.

After finishing the long courtship echeme, a male produces a very conspicuous display with his long antennae (the ratio of antennae length to head and pronotum length averages 1.89±0.07 in *C.macrocerus* in contrast to 1.64±0.1 in *C.apricarius* or 1.70±0.17 in *C.fallax*). First, antennae are moved in longitudinal plane backwards, and the two antennae are moved asymmetrically (Fig. 8A). Then, antennae are moved in horizontal plane, from side to side (Fig. 8B) and finally, antennae are moved in a circular manner (Fig. 8C). Immediately after the antennal movements (see Suppl. material 4), a male is trying to mate. We documented the antennal movements in the only males from Georgia (nominative subspecies). Concerning the males from other localities (*C.macroceruspurpuratus*), we cannot say whether they show such visual display or not, since we did not pay attention to the antennal movements.

#### Comparative remarks.

The current recordings of calling song in *C.macroceruspurpuratus* are similar to the previous recordings. Vedenina and Bukhvalova (2001) showed that in two subspecies, *C.macroceruspurpuratus* and *C.macrocerusponticus*, the echeme duration varies greatly (in the range of ~ 3.5–15 s); however, it varies in different specimens referred to the same subspecies. On the basis of this similarity and similarity in morphology between *C.macroceruspurpuratus* and *C.macrocerusponticus*, the authors doubt whether these subspecies should be distinguished. By contrast, nominative subspecies differs from *C.macroceruspurpuratus* by some morphological characters (the body and wing lengths are larger in nominative subspecies) and some song parameters (the echeme duration is higher and the syllable rate is less in nominative subspecies).

The leg-movement and song patterns in *C.macrocerus* are relatively simple and may be considered as plesiomorphic (Vedenina and Mugue 2011; Sevastianov et al. 2023). Moreover, it was suggested that the calling and courtship songs are similar in this species. Therefore, our discover of specific movements with antennae during courtship in *C.macrocerus* is remarkable. In future, it would be important to study if the males in *C.macroceruspurpuratus* demonstrate similar movements with antennae as it was found in nominative subspecies. Considering the very long antennae typical for this species, we expect similar visual display in different subspecies.

### ﻿ Chorthippus hammarstroemi

Taxon classificationAnimaliaOrthopteraAcrididae

(Miram, 1907)

87437CCA-B3CB-5CA6-9C95-F50B3D387206

#### Distribution.

Southern Siberia from Altai to Transbaikalia, southern part of the Russian Far East, Mongolia, China.

#### Material

**(Fig. 1).** Russia: 14. Altai Republic, Chemal district, Elekmonar, 51°27.372'N, 86°02.524'E, 459 m a.s.l., 03.08.2023, song recordings in 2 ♂; 15. Altai Republic, Ongudai district, ab. 3.5 km S of Inya, 50°24.840'N, 86°38.120'E, 769 m a.s.l., 05.08.2023, song recordings in 6 ♂.

#### References to song.

Benediktov 2005: recordings of calling song from Tuva; Tishechkin and Bukhvalova 2009: recordings of calling and courtship songs from Buryatia, Chita region and Maritime Province.

#### Song.

**Figure 9.**
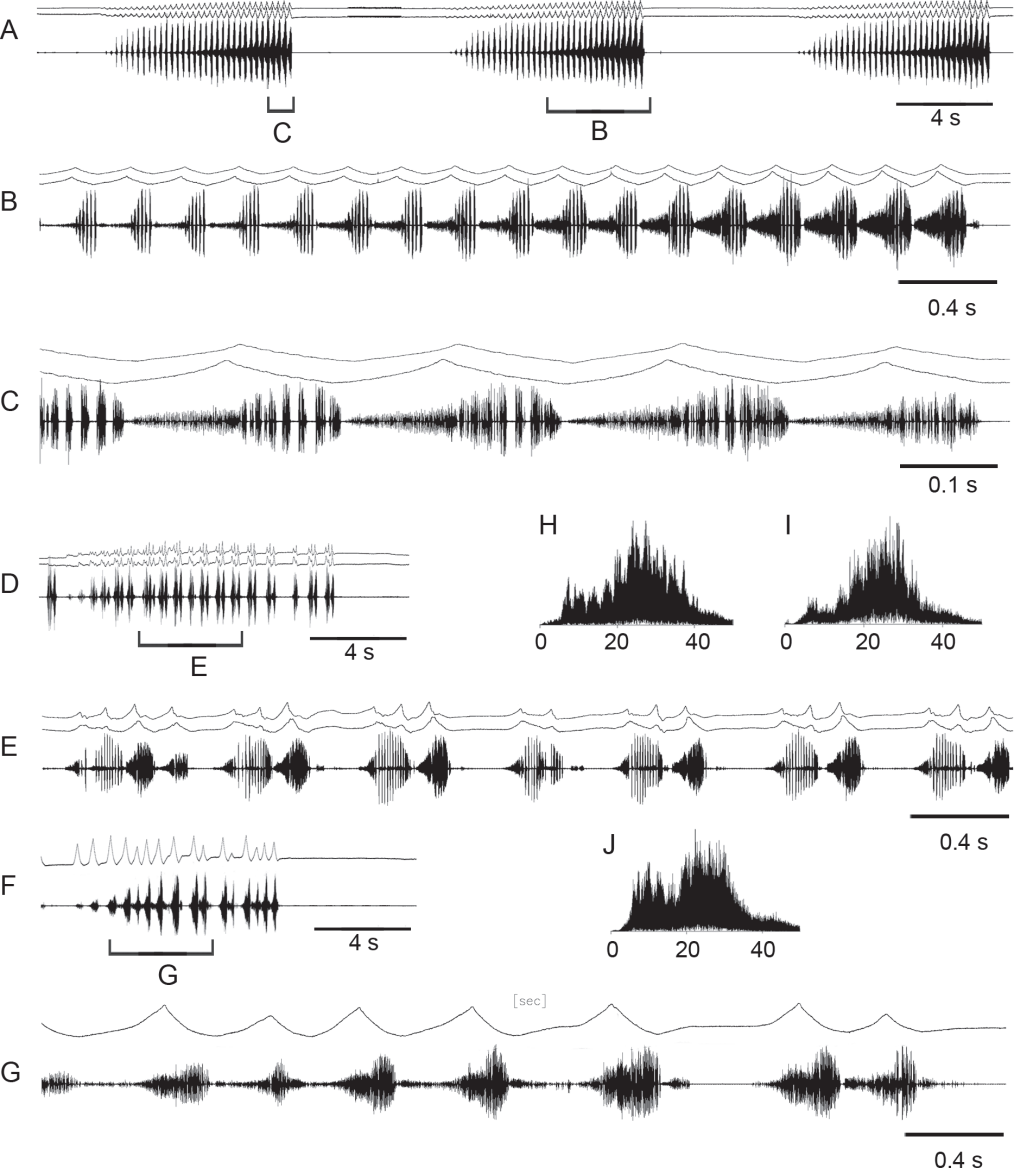
Oscillograms of the calling song **A–C** rivalry song **D–E** courtship song **F–G** and frequency spectra **H–J** in *Chorthippushammarstroemi*. Song recordings are presented at three different speeds. In all oscillograms except **F, G** the two upper lines are recordings of hind leg movements and the lower line is the sound recording. In **F, G** the movements of only one leg are shown. Frequency spectra are shown in kHz for the calling song **H** rivalry song **I** and courtship song **J**.

The calling song is an echeme of variable duration ranging from ~ 6 to 20 s. Sometimes a male can produce several echemes with intervals of ~ 4–6 s (Fig. 9A). The legs being moved with a small phase shift at the rate of ~ 4–4.5 /s generate syllables where one can distinguish soft and loud parts (Fig. 9B, C). During each upstroke, almost no (in the beginning of echeme) or relatively soft (in the end of echeme) sound is produced; during downstroke, a loud sound is generated. The oscillograph analysis shows that the legs slightly vibrate during downstroke, which result to producing distinct pulses, especially at the beginning of echeme.

The rivalry song is a sequence of short echemes (Fig. 9D), which number can greatly vary. Each echeme consists of two or three syllables. The legs are moved almost synchronously at the rate of ~ 7.5 /s. The first syllable usually contains distinct pulses that are similar to those in the calling song. In the noisy second and third syllables the pulses are not distinguishable (Fig. 9E).

A courted male generates an echeme or several echemes that are similar to the calling song. The legs are moved at the slightly higher rate than during calling (of ~ 5–6 /s). After this, a male can produce noisy syllables by the high-amplitude synchronous leg movements at the rate of ~ 2–3 /s (Fig. 9F, G). Before copulation attempt, males move with the long antennae (the ratio of antennae length to head and pronotum length averages 2.07±0.14). The movement pattern is simpler than in *C.macrocerus*: antennae in *C.hammarstroemi* are only moved in a circular manner as in Fig. 8C. Sometimes, the courting male produces the song similar to rivalry song, and the short echemes containing two types of syllables (as in Fig. 9E) can alternate with the noisy courtship element (as in Fig. 9G).

The power spectra of the calling and rivalry songs are similar and have maximum energy between 20 and 35 kHz (Fig. 9H, I). The power spectrum of specific courtship element has two broad peaks, between 5 and 15 kHz and between 20 and 30 kHz (Fig. 9J).

#### Comparative remarks.

The current recordings from Altai are similar to the previous recordings from the more eastern localities of this species (Benediktov 2005; Tishechkin and Bukhvalova 2009). We, however, found slight differences in courtship songs between specimens from Altai and those from Buryatia, Chita region, and Maritime Province (Tishechkin and Bukhvalova 2009). At the same time, considering variable courtship behaviour and different technique of sound recordings, we suggest no principal differences between the recordings.

By contrast, the antennal movements in *C.hammarstroemi* during courtship are documented for the first time. This species is similar to *C.macrocerus* by the remarkably long antennae. The two species are also similar in plesiomorphic pattern of the leg movements during calling behaviour. Tishechkin and Vedenina (2016) suggested that these two allopatric species are characterised by similar song type and acoustic strategy. The usage of long antennae in courtship behaviour in both species even confirms such similarity. On the other hand, both morphological (Bey-Bienko and Mistshenko 1951) and molecular studies (Sevastianov et al. 2023) suggest that *C.macrocerus* and *C.hammarstroemi* are not closely related species. We suggest one of the reasons of their allopatric distribution to be a similarity of acoustic niches, which prevents them from effectively finding members of their own species at the same biotope (Bukhvalova and Zhantiev 1994; Tishechkin and Vedenina 2016).

### ﻿ Megaulacobothrus aethalinus

Taxon classificationAnimaliaOrthopteraAcrididae

(Zubowsky, 1899)

01564E8C-AA9F-5CAD-8D32-2377450ED86F

#### Distribution.

Southern Siberia, the southern part of Russian Far East, the north-eastern China, Korea.

#### Material

**(Fig. 1).** Russia: 10. Altai Republic, ab. 6 km of Chemal, environs of Elekmonar, 51°29.0'N, 85°59.9'E, 417 m a.s.l., 06.08.2017, song recordings in 2 ♂, 12.08.2021, song recordings in 4 ♂; 11. Altai Republic, Ongudai district, ab. 7 km N of Malyi Yaloman, 50°33.602'N, 86°33.783'E, 740 m a.s.l., 09.08.2023, song recordings in 1 ♂; 12. Altai Republic, Ongudai district, near Shirlak waterfall, 50°20.6'N, 87°13.3'E, 1064 m a.s.l., 14.08.2021, song recordings in 3 ♂; 13. Altai Republic, Ongudai district, ab. 15 km NWW of Chibit, 50°21.637'N, 87°19.480'E, 1056 m a.s.l., 09.08.2023, song recordings in 1 ♂.

#### References to song.

Bukhvalova and Vedenina 1998; Vedenina and Bukhvalova 2001; Benediktov 2005: recordings of calling song from Altai and Maritime Province.

#### Song.

**Figure 10.**
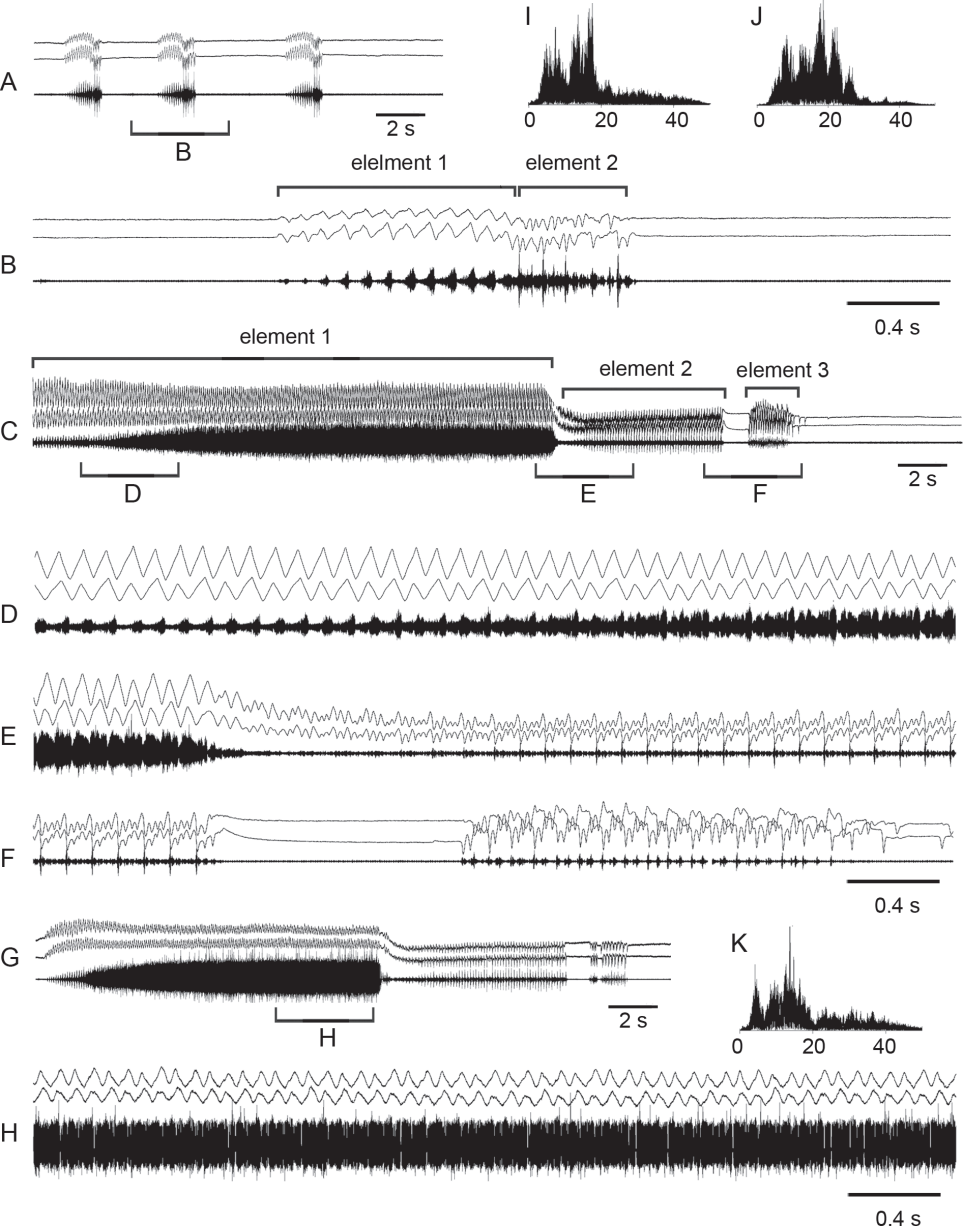
Oscillograms of the calling song **A, B** courtship songs **C–H** and frequency spectra **I–K** in *Megaulacobothrusaethalinus*. Courtship songs of two males are shown in **C** and **G**. Song recordings are presented at two different speeds. In all oscillograms the two upper lines are recordings of hind leg movements and the lower line is the sound recording. Different elements of the calling song are shown in **B** of the courtship song – in **C**. Frequency spectra are shown in kHz for the calling song **I** for the courtship element 1 **J** and courtship element 2 **K**.

The calling song is a sequence of several echemes lasting ~ 1.5–3 s and separated by intervals of ~ 2–6 s (Fig. 10A). In each echeme, one can distinguish two parts (see Suppl. material 5). In the first part, the legs are moved synchronously at the rate of 7.5–10 /s, which result to generation of simple regular syllables (element 1, Fig. 10B). The sound is generated mainly during downstroke. In the second part, the legs are moved asynchronously, which results to generation of the louder element 2 containing pulses of varying amplitude. The duration of element 2 is usually 2× as short as duration of element 1.

The courtship song starts similarly to the first part of the calling song: the legs are moved synchronously and generate the soft sound during upstroke and the louder sound during downstroke (Fig. 10C, D). In ~ 2–10 s, however, the temporal structure of syllables is gradually changing. The intervals between syllables become fuzzy and the very syllables become louder. Sometimes, the intervals between syllables become completely indistinguishable (Fig. 10H). This main echeme (element 1 of courtship) usually lasts for ~ 15–30 s, but can continue for more than 1 min. Oscillographic analysis of the leg movements shows that amplitude of the two neighbouring strokes slightly differs, however, this is more expressed in one leg (Fig. 10D, E). After the prolonged element 1, a much shorter (lasting ~ 6–9 s) element 2 follows. The legs are moved synchronously but at the lower position, and the leg movements have the more complex pattern than during element 1, which implies alternation of several low-amplitude strokes with one higher-amplitude stroke. The legs generate syllables repeated at the rate of 8–10 /s (Fig. 10E); each syllable contains one high-amplitude pulse and several soft pulses. In ~ 1 s after the end of element 2, the shortest (lasting ~ 1–2 s) element 3 follows (Fig. 10C, F, G; see Suppl. material 6). The leg movements have the more irregular pattern than during element 2, moreover, the two legs are moved alternately. As a result, one can distinguish sound pulses repeated at the rate of ~ 15–20 /s.

The frequency spectra of both types of the song are remarkable because they occupy the band lower than 25 kHz. The spectrum of the calling song has two peaks around 7 and 18 kHz (Fig. 10I), the spectrum of the courtship element 1 has several peaks in the range from 5 to 25 kHz (Fig. 10J), and the spectrum of the courtship element 2 has two peaks at approximately 5 and 15 kHz (Fig. 10K).

**Figure 11.**
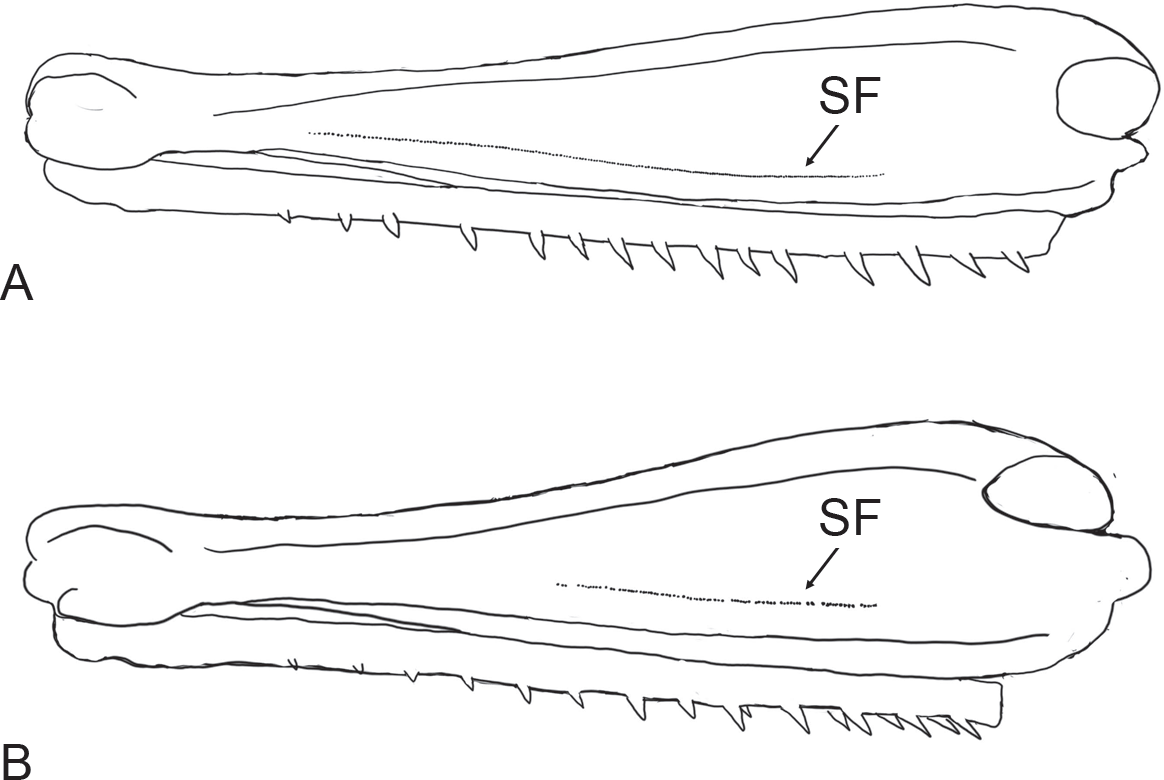
Stridulatory files SF on the hind femora in *Megaulacobothrusaethalinus***A** and *Chorthippusmacrocerus***B**.

The elements 1 and 2 of the calling song and the elements 1–3 of the courtship song are produced at the different leg positions. During generation of the calling element 1 and the courtship elements 2 and 3, the legs are moved at the low position. Presumably the distal stridulatory pegs of the hind femora are used in generation of these elements. We compared the lengths of the stridulatory files between *M.aethalinus* and *C.macrocerus*, the species with the simpler song. The file length appeared to be almost 2× longer in *M.aethalinus* than that in *C.macrocerus*. In *M.aethalinus*, the most distal stridulatory pegs are at about the level of the first tibial spine if tibia is pressed to femur (Fig. 11A). In *C.macrocerus*, the most distal stridulatory pegs are at the level between 5^th^ and 6^th^ tibial spines (Fig. 11B).

#### Comparative remarks.

Our recordings of both types of the song are generally similar to those previously described by different authors (Vedenina and Bukhvalova 2001; Benediktov 2005). However, previous authors argued about the functions of the two song types in *M.aethalinus*. According to Vedenina and Bukhvalova (2001), different song types are produced by a male sitting alone, therefore they can be considered as the calling song variants. Benediktov (2005) suggested the first song type to be the calling song and the second song type to be the courtship song. Our current data are more concordant with the data of Benediktov (2005). However, our observations of behaviour in this species show that the males sitting without females in laboratory for several days start to produce both song types almost equally often. In nature, by contrast, solitary males usually sing the first song type, whereas the males sitting near by the females typically produce the second song type. Therefore, we also assign the different functions to the different song types.

Analysis of the elaborate leg movements during stridulation in *M.aethalinus* allowed us to suggest unusually long stridulatory file. Up to now, only several species of *C.biguttulus* group with the long stridulatory files were known (Benediktov 1999; Willemse et al. 2009; Tarasova et al. 2021). One of these species, *C.biguttulusehedickei*, also uses its distal part of the stridulatory file in generation of a relatively soft ‘aftersong’. *M.aethalinus*, however, produces a relatively loud sound with the distal part of the stridulatory file.

## ﻿Conclusions

1. In seven species of subfamily Gomphocerinae, the stridulatory leg movements were recorded and analysed for the first time. In *Mesasippuskozhevnikovi*, *Chorthippusmacrocerus* and *C.hammarstroemi*, the legs are moved in a relatively simple pattern that is considered to be plesiomorphic (Vedenina and Mugue 2011; Sevastianov et al. 2023). Other four species, *Myrmeleotettixpalpalis*, *Stenobothrusnewskii*, *C.pullus*, and *Megaulacobothrusaethalinus* demonstrate more complex leg movements, which are considered to be the more evolutionary advanced patterns.

2. The number of sound elements in the calling and courtship songs is the same in *C.macrocerus*. The courtship song contains one additional sound element in *S.newskii*, *M.kozhevnikovi*, *C.pullus*, and *C.hammarstroemi*. The highest number of courtship sound elements is found in *M.palpalis* and *M.aethalinus*.

3. The songs in *S.newskii* are shown for the first time. This species is remarkable by crepitation in flight and generation of short wing beats, which brings this species closer to other three species of *Stenobothrus* (*S.rubicundulus*, *S.cotticus*, and *S.hyalosuperficies*). Moreover, we found a high similarity between *S.newskii* and *S.cotticus* in acoustic behaviour, morphology and ecological preferences, which may indicate that these species belong to the same taxon. However, a large distance between habitats of these species do not allow us to make final conclusions.

4. The courtship songs in two species, *M.palpalis* and *M.aethalinus*, contain several sound elements. The complexity of the courtship song in *M.palpalis* is in a concordance with the complexity of courtship behaviour in other species of the genus *Myrmeleotettix.* The song complexity in *M.aethalinus* stands apart because it is not typical for the tribe Gomphocerini. The different courtship song elements in *M.aethalinus* are produced by vibrating hind femora at the different positions. Analysis of the leg movements revealed the participance of different parts of the long stridulatory file in sound production.

5. A maximum energy of the song power spectra in 6 species studied lies in ultrasound range (higher than 20 kHz). In only *M.aethalinus*, the main peaks in the song power spectra lie lower than 20 kHz. This should be considered during analysis of the recordings made by portable recorders with a frequency range not exceeding 12.5–15 kHz.

6. The courtship behaviour in *M.palpalis*, *C.macrocerus*, and *C.hammarstroemi* includes a different visual display. For the first time we documented conspicuous movements with long antennae in *C.macrocerus* and *C.hammarstroemi*, which are demonstrated just before a copulation attempt. We suggest a correlation between the antenna length and the antennal movements during courtship. *M.palpalis* shows slight movements with antennae and the whole body, and very conspicuous movements with palps during courtship, which are very different from those in other species of the genus *Myrmeleotettix*.

## Supplementary Material

XML Treatment for Myrmeleotettix palpalis

XML Treatment for Stenobothrus newskii

XML Treatment for Mesasippus kozhevnikovi

XML Treatment for Chorthippus pullus

XML Treatment for Chorthippus macrocerus

XML Treatment for Chorthippus hammarstroemi

XML Treatment for Megaulacobothrus aethalinus

## ﻿Additional information

### Author ORCIDs

Varvara Vedenina https://orcid.org/0000-0002-2694-4152

Nikita Sevastianov https://orcid.org/0000-0002-1563-5194

### Data availability

All of the data that support the findings of this study are available in the main text or Supplementary Information.

## Supplementary materials

Supplementary material 1 Courtship in *Myrmeleotettixpalpalis* Data type mpg Explanation note The male produces high-amplitude strokes with hind legs, moves his body from side to side, generates low-amplitude movements with antennae, and conspicuously moves with palps up and down. In this movie and some others, the pieces of reflecting foil glued to the distal part of each hind femur remained from the leg-movement recordings. https://binary.pensoft.net/file/1040941This dataset is made available under the Open Database License (http://opendatacommons.org/licenses/odbl/1.0/). The Open Database License (ODbL) is a license agreement intended to allow users to freely share, modify, and use this Dataset while maintaining this same freedom for others, provided that the original source and author(s) are credited.Varvara Vedenina, Nikita Sevastianov, Evgenia Kovalyova

Supplementary material 2 Courtship in *Stenobothrusnewskii* Data type mpg Explanation note The male generates sound by both femoral-tegminal stridulation and wing clapping. The female responds by stridulation. The male attempts to copulate. https://binary.pensoft.net/file/1040942This dataset is made available under the Open Database License (http://opendatacommons.org/licenses/odbl/1.0/). The Open Database License (ODbL) is a license agreement intended to allow users to freely share, modify, and use this Dataset while maintaining this same freedom for others, provided that the original source and author(s) are credited.Varvara Vedenina, Nikita Sevastianov, Evgenia Kovalyova

Supplementary material 3 Courtship in *Chorthippuspullus* Data type mpg Explanation note The first whizzing element is produced by the low-amplitude leg movements, after which the legs are moved asynchronously with the high amplitude, which produces almost no sound. https://binary.pensoft.net/file/1040943This dataset is made available under the Open Database License (http://opendatacommons.org/licenses/odbl/1.0/). The Open Database License (ODbL) is a license agreement intended to allow users to freely share, modify, and use this Dataset while maintaining this same freedom for others, provided that the original source and author(s) are credited.Varvara Vedenina, Nikita Sevastianov, Evgenia Kovalyova

Supplementary material 4 Courtship in *Chorthippusmacrocerus* Data type mpg Explanation note Immediately after the song generation, the male conspicuously moves with antennae and attempts to copulate. https://binary.pensoft.net/file/1040944This dataset is made available under the Open Database License (http://opendatacommons.org/licenses/odbl/1.0/). The Open Database License (ODbL) is a license agreement intended to allow users to freely share, modify, and use this Dataset while maintaining this same freedom for others, provided that the original source and author(s) are credited.Varvara Vedenina, Nikita Sevastianov, Evgenia Kovalyova

Supplementary material 5 Calling in *Megaulacobothrusaethalinus* Data type mpg Explanation note The male produces two elements, one element when the legs are maintained at the higher position, the second element when the legs are kept at the lower position. https://binary.pensoft.net/file/1040945This dataset is made available under the Open Database License (http://opendatacommons.org/licenses/odbl/1.0/). The Open Database License (ODbL) is a license agreement intended to allow users to freely share, modify, and use this Dataset while maintaining this same freedom for others, provided that the original source and author(s) are credited.Varvara Vedenina, Nikita Sevastianov, Evgenia Kovalyova

Supplementary material 6 Courtship in *Megaulacobothrusaethalinus* Data type mpg Explanation note Three elements of the courtship song are produced: element 1 is produced by synchronous leg movements kept at the higher position, element 2 is produced by also synchronous leg movements but kept at the lower position, the short element 3 is produced by asynchronous leg movements. https://binary.pensoft.net/file/1040946This dataset is made available under the Open Database License (http://opendatacommons.org/licenses/odbl/1.0/). The Open Database License (ODbL) is a license agreement intended to allow users to freely share, modify, and use this Dataset while maintaining this same freedom for others, provided that the original source and author(s) are credited.Varvara Vedenina, Nikita Sevastianov, Evgenia Kovalyova
